# Investigation of Long- and Short-Term Relationships Between Cesarean Delivery and Its Effective Factors in Malayer

**DOI:** 10.5539/gjhs.v6n7p1

**Published:** 2014-09-18

**Authors:** V. Alinejad, M. Mahmodi, M. Alinejad, E. Besharat, R. Gholizade, E. Tabbakhi, A. Shojaei Pour, R. Gharaaghaji

**Affiliations:** 1 Center of Clinical Research and Reproductive Health, Urmia University of Medical Sciences, Urmia, Iran; 2 Bio-Statistics and Epidemiology Group of Hamedan Faculty of Medicine, Iran; 3 Tabriz University of Medical Sciences, Tabriz, Iran; 4 Urmia University, Iran; 5 Educational Management, Urmia University of Medical Sciences, Urmia, Iran; 6 Social Science Research, Urmia University of Medical Sciences, Urmia, Iran; 7 Department of Fars Education, Shiraz, Iran; 8 Cellular and Molecular Research Center, Urmia University of Medical Sciences, Urmia, Iran

**Keywords:** short-term relationship, long-term relationship, cesarean

## Abstract

**Introduction:**

Recently, there has been significant increase in the number of operated cesarean compared to the overall number of birth giving. There are several factors affecting the operated cesarean in Iran compared to the birth giving which are to be reviewed in this study.

**Procedure:**

The data of the study has been obtained from the registered information in Assistance Section of Health at Hamedan Faculty of Medicine which includes the seasonal data having to do with giving birth of Malayer since the beginning of Winter 2006 to the end of Fall 2013. The assimilation techniques, namely ARDL method and Error Correction Method (ECM) are the main methods to be used in this study.

**Results:**

The short-term and long-term coefficients of abnormal view, incongruent status of fetus and pelvis, lack of progression, and the lengthy status are considered significant statistically. The ecm coefficient is -1.3456 in short-term. Also, his coefficient is significant which shows the short-term balance trend to the long-term one.

**Conclusion:**

The most indispensable affective factor on demanding to run the cesarean operation in short-term and long-term in Malayer are the lengthy-status, lack of progression, abnormal view, and incongruent status of fetus and pelvis, respectively.

## 1. Introduction

The increasing trend of cesarean deliveries is a major concern for the medical society as well as the health authorities in Iran. In cases where natural vaginal delivery is not possible, including abnormal presentation, placenta previa, dystocia, post-term pregnancy, repeat cesarean, cord prolapsed, and multiple birth, delivery should be carried out through cesarean section (Lee-Parritz, 2004; Cunningham, 2005). However, unnecessary cesarean surgeries are accompanied by considerable costs and complications, such as bleeding, infection, and infertility, compared to natural vaginal delivery (Duff, 2010). According to DHS, the rate of cesarean deliveries has been increasing in both developing and developed countries in the recent years (Najmi & Rehan, 2000). The studies conducted in England, U.S., and South America has also shown the growing trend of cesarean deliveries (Dosa, 2001; Leitch & Walker, 1998; Menacker & Curtin, 2001; Gomes et al., 1999; Flamm, Berwick, & Kabcenell, 1998). Although some countries have succeeded in controlling the cesarean deliveries (Flamm et al., 1998; Notzon et al., 1994), a large number of deliveries are carried out through cesarean surgeries in a large number of countries (Menacker & Curtin, 2001; Wilkinson et al., 1998). Moreover, the results of a study performed in England have indicated that the risk of maternal mortality is 3 times higher in cesarean section compared to natural delivery (Dosa, 2001).

In spite of the great attempts in Iran, this country is still faced with women’s lack of knowledge regarding the advantages and disadvantages of both natural and cesarean deliveries. Most pregnant women consider cesarean section as a safe delivery mode. According to the statistics, the rate of cesarean deliveries in Iran is quite higher than the global standard, leading to a great concern for the authorities of medical society. Hamadan province is also faced with this problem; such a way that the rate of cesarean deliveries increased by 4.3% since 2009 to 2011. Hence, despite the policies of the healthcare system, the increasing trend of cesarean deliveries necessitates performance of further investigations and presentation of more effective strategies. In fact, prediction and assessment of the role of the effective factors in cesarean delivery can guide the society towards controlling the growing trend of cesarean surgeries which eventually reduces the negative consequences of selection of the inappropriate delivery mode. The present study aims to model delivery and the factors affecting it using Autoregressive Distributed Lag (ARDL) time series models. In comparison to other statistical methods, not only time series models assess the role of each effective factor in cesarean delivery, but they also evaluate and predict its trend which can be effective in correct planning for reduction of unnecessary cesarean surgeries in future.

## 2. Materials and Methods

The present study is a cross-sectional investigation of all the deliveries and cesarean sections in Malayer. Malayer is located in the southeast of Hamadan province, Iran. The area of this city is 3210 km^2^ and it consists of 8 districts and 220 villages. In 2011, the population of Malayer was 287982 individuals, with women comprising the largest part of the population. Malayer is the second large city of Hamadan province after Hamadan (Website of the Statistical Center of Iran, 2011). The data of the present study included the seasonal data of delivery status in Malayer from the beginning of winter of 2006 to the end of autumn of 2012. The data were obtained from the information that the hospitals of Malayer provided to the health deputy of Hamadan University of Medical Sciences, Hamadan, Iran. In this study, the factors affecting cesarean delivery were abnormal presentation (all fetal presentations, except for head presentation, including transverse, face, chin, and breech presentations), cephalopelvic disproportion (resulting from decreased pelvic volume, large baby, or both), post-term pregnancy (pregnancy after the 42nd week after the first day of the last menstrual cycle), and dystocia (any factor which lengthens or disrupts the process of delivery, including pelvic dysfunction, pelvic disorders, soft tissue disorders, etc.). In order to analyze the data, time series models using co-integration techniques, particularly ARDL and Error Correction Method (ECM), were utilized in the present study. The study data included all the referrals for cesarean delivery in the previous 24 seasons as well as the reasons for selection of this mode in the hospitals of Malayer. The time series model for the trend of the performed cesarean deliveries in Malayer is as follows: (Kaveh & Dalfard, 2012)


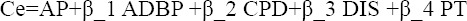


Where Ce is the logarithm of cesarean demand volume, ADBP is the logarithm of abnormal presentation, CPD is the logarithm of cephalopelvic disproportion, DIS is the logarithm of dystocia, and PT is the logarithm of post-term pregnancy. Estimation of the parameters and analysis of the designed dynamic models were performed using Microfit 4.1 and EVies 6 software.

## 3. Results

In the current study, augmented Dickey-Fuller test was used in order to evaluate stationarity and existence of unit root. In case of significance, existence of unit root and stationarity are rejected. Thus, the critical quantity at level condition, with no trend, and CI=95% was -2.998. The P-values of Dickey-Fuller test have been presented in Table 1. According to the results, Ce, ADBP, and PT were non-stationary.

**Table 1:** The results of augmented Dickey-Fuller test for the model variables at level condition (with intercept, without trend)

Variable	Number of lags	t in augmented Dickey-Fuller test	P-value
Ce	1	-4.31	0.0028
ADBP	0	-4.59	0.0015
CPD	0	-1.12	0.6881
DIS	1	-1.43	0.5525
PT	0	-5.49	0.0002

ADF=(-2.998) at 5% level.

As Table 2 depicts, the augmented Dickey Fuller test statistic became larger than the critical quantity after first-order differencing the variables and, consequently, the stationarity of the variables was proved. Therefore, cesarean, abnormal presentation, and dystocia were stationary at level condition, while cephalopelvic disproportion and post-term pregnancy were stationary at first order.

**Table 2:** The results of augmented Dickey-Fuller test for the model variables at first-order difference (with intercept, without trend)

Variable	Number of lags	t in augmented Dickey-Fuller test	P-value
CPD	0	-7.57	0.000
PT	1	-4.62	0.0015

ADF=(-3.004) at 5% level.

***Estimation of cesarean demand function using ARDL***: In order to determine the long-term relationships and co-integration analyses, single-equation method by Pesaran and Shin (1997) and Pesaran et al. (1977) was utilized. In the method proposed by Pesaran and Shin, the estimation process is performed through two stages. The first stage which is the prerequisite to co-integration methods is the stationarity of the variables. The superiority of ARDL to other co-integration methods is that it can estimate both short- and long-term relationships in case the model variables are non-zero-order stationary or first-order stationary. In addition, it provides quite efficient estimations (Pesaran & Smith, 1998). The second stage involves estimation of short- and long-term relationships. The results of estimation of cesarean demand function have been presented in the following table.


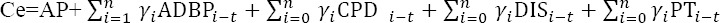


Considering P=0.093 for autocorrelation test, assumption of H0 based on lack of autocorrelation is confirmed. In addition, based on P=0.065 in the true function test, assumption of H0 based on the true function is confirmed. Besides, considering P=0.663 in the normality test, assumption of H0 based on normality is confirmed. Based on P=0.115 in variance anisotropy test, assumption of H0 based on variance anisotropy is also confirmed. Hence, the model is confirmed to be an appropriate one.

***Co-integration test:*** In order to determine long-term convergence, the amount of *t* is computed by the following equation:


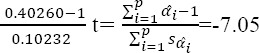


By comparison of the calculated value to the critical quantity presented by Banerjee, Dolado, and Mastre at 90% confidence level, assumption of H0 based on lack of long-term convergence among the model variables was rejected, and a long-term equilibrium relationship among the variables was confirmed.

***Long-term analysis of the function of demands for cesarean section:*** In this section, the long-term relationship among the variables was assessed using the coefficients of dynamic ARDL model.

Due to the fact that a logarithmic model was employed in the present study, the coefficients of the variables represent the elasticity of abnormal presentation, cephalopelvic disproportion, dystocia, and post-term pregnancy. Based on Table 5, long-term elasticity of the total demand for cesarean relative to abnormal presentation was -7.3669. This implies that by 1% increase (decrease) in the rate of abnormal presentation in long term, the rate of cesarean section increased (decreased) by 7.3669%. This was statistically significant, indicating the effectiveness of changes in the rate of abnormal presentation in the rate of total cesarean demand. Moreover, the long-term elasticity of the total demand for cesarean section relative to cephalopelvic disproportion was 3.8599. Thus, 1% increase (decrease) in the rate of cephalopelvic disproportion led to 3.8599% increase (decrease) in the total demand for cesarean delivery. Furthermore, long-term elasticity of the total demand for cesarean section relative to dystocia was 9.5491. This shows that by 1% increase (decrease) in the rate of dystocia in long term, 9.5491% increase (decrease) could be observed in the rate of total demand for cesarean delivery. This was statistically significant, revealing the effectiveness of changes in the rate of dystocia in the total demand for cesarean section. Finally, post-term pregnancy was also significantly effective in the rate of total demand for cesarean delivery. According to Table 5, by 1% increase (decrease) in the rate of post-term pregnancy, 44.3474% increase (decrease) could be observed in the rate of total demand for cesarean delivery.

**Table 3:** The results of estimation of cesarean demand function using ARDL (1, 0, 0, 1, 0)

Variable	Coefficient	SD	P-value
Ce (-1)	-0.34557	0.19073***	0.088
ADBP	-9.9124	5.3874***	0.083
CPD	5.1937	1.0185*	0.000
DIS	6.0084	3.0708***	0.067
DIS (-1)	6.8406	3.0018**	0.036
PT	59.6724	28.55***	0.052

F=4.2974, R^2^=0.5583.

*, **, and *** represent significance level at 1%, 5%, and 10%, respectively.

**Table 4:** The results of long-term estimation of function of demand for cesarean using ARDL (1, 0, 0, 1, 0)

Variable	Coefficient	SD	P-value
ADBP	-7.3669	4.1738***	0.096
CPD	3.8599	0.70098*	0.000
DIS	9.5491	1.8659*	0.000
PT	44.3474	20.3675**	0.044

*, **, and *** represent significance level at 1%, 5%, and 10%, respectively.

**Table 5:** The results of short-term estimation of function of demand for cesarean using ECM

Variable	Coefficient	SD	P-value
d ADBP	-9.9126	5.3874***	0.082
d CPD	5.1937	1.0185*	0.000
d DIS	6.0084	3.0708***	0.066
d PT	59.6724	28.5500***	0.051
ecm (-1)	-1.3456	0.19073*	0.000

F=13.7945, R^2^=0.76447.

*, **, and *** represent significance level at 1%, 5%, and 10%, respectively.

***Short-term analysis of demands for cesarean delivery using ECM:*** This section deals with the short-term analysis of the function of demand for cesarean delivery. The coefficients of ECM which represent the relationships between cesarean section and independent variables in short terms have been presented in Table 5.


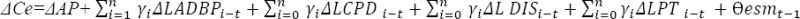


The estimated coefficients represent short-term elasticity of the rate of demand for cesarean delivery relative to abnormal presentation, cephalopelvic disproportion, dystocia, and post-term pregnancy. According to the results, 1% increase (decrease) in the rate of abnormal presentation resulted in 0.9126% decrease (increase) in the total demand for cesarean delivery. Moreover, 1% increase (decrease) in the rate of cephalopelvic disproportion led to 5.1937% increase (decrease) in the total demand for cesarean delivery, which was statistically significant. Additionally, by 1% increase (decrease) in the rate of dystocia, the total demand for cesarean delivery increased (decreased) by 6.0084%. Also, by 1% increase (decrease) in the rate of post-term pregnancy, 59.6724% increase (decrease) could be observed in the total demand for cesarean delivery, which was statistically significant. In this study, ECM (-1) coefficient was -1.3456 in short term, which was statistically significant. This revealed the speed of short-term adjustment towards long-term adjustment. In fact, this coefficient showed that 1.3456% of imbalance in each period is adjusted in the next one. Besides, R^2^ was 76.447% in short term which implies that almost 76.447% of the changes in the total demand for cesarean section in short term is described by the model’s explanatory variables. Furthermore, highness of both F and R_2_ confirm the appropriateness of the model.

***Evaluation of the stationarity of CUSUM and CUSUMQ coefficients:*** This test has been suggested for investigation of stationarity of the model coefficients. In this method, first a regression equation including the intended variables is estimated by the minimum estimable observations. Afterwards, an observation is added to the previous equation and estimation is performed again and the process continues. In this way, after estimation of each stage, a coefficient is obtained for each variable, eventually resulting in a time series of variables coefficients. In case the model is stationary, the changes in the coefficients are expected to be slight and random.

**Graph 1:**
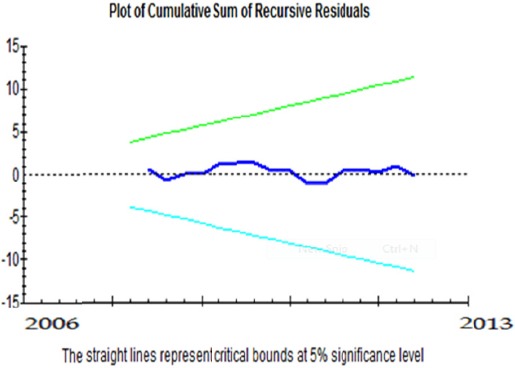
Assessment of the stationarity of short-term coefficients

**Graph 2:**
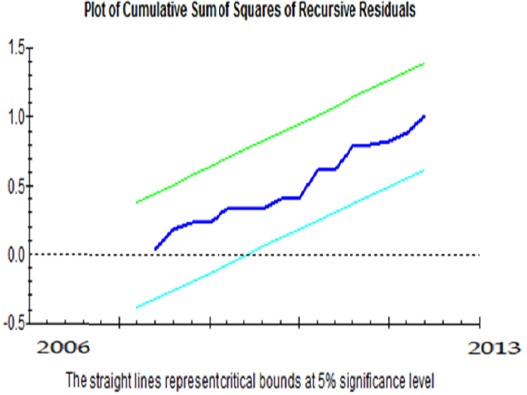
Assessment of the stationarity of long-term coefficients

The results of these tests which have been presented in Graphs 1 and 2 indicated the stationarity of the estimated coefficients of short- and long-term models. It should be mentioned that the straight lines indicate significance at 5% level.

## 4. Conclusions

Modeling the estimation of function of demand for cesarean delivery in short and long term showed that the cross-elasticity of post-term pregnancy was higher than the elasticity of abnormal presentation, cephalopelvic disproportion, and dystocia. Hence, the most effective factors in demand for cesarean delivery in Malayer in short and long term were post-term pregnancy, dystocia, abnormal presentation, and cephalopelvic disproportion. According to the statistics, post-term pregnancies comprise 5-10% of all pregnancies (Zeitlin, 2007). Studies have shown that post-term pregnancy not only increases the risk of prenatal mortality, but is also accompanied by the risk of delivery complications for the mother (Hilder et al., 1998; Lindstrom et al., 2005). However, a limited number of studies have investigated the causes of post-term pregnancy. Yet, some risk factors have been identified. For instance, a study which was conducted in Sweden revealed that as the mother’s age increased, the risk of post-term pregnancy increased, as well (Roos et al., 2010). Thus, educational programs should be planned and necessary measures should be taken in order to reduce pregnancy in higher ages. Furthermore, one study indicated that high fetal weight led to dystocia (Mollberg et al., 2005). By reducing the post-term pregnancies, high fetal weight can be prevented, consequently decreasing the rate of dystocia and cesarean sections. The results of the present study are consistent with the results of our study (Alinejad et al., 2013). Overall, the findings of the present study showed that post-term pregnancy was quite more important compared to other factors. In general, in case delivery cannot be carried out through the natural route, cesarean section can be selected for reducing the mortality. Nevertheless, by decreasing the indications of cesarean delivery, the rate of this surgery can be reduced. Also, by planning and providing the pregnant women with the knowledge regarding the benefits and disadvantages of natural and cesarean deliveries, the rate of unnecessary elective cesarean sections can be decreased. In this way, the negative consequences of cesarean surgeries and their extra costs can be considerably reduced.
